# Prognostic significance of the neutrophil-to-lymphocyte ratio in peripheral T-cell lymphoma: a meta-analysis

**DOI:** 10.1186/s12935-021-02391-z

**Published:** 2021-12-19

**Authors:** Jia Liu, Shengnan Zhang, Ruihua Mi, Lin Chen, Qingsong Yin

**Affiliations:** 1 Department of Hematology, The Affiliated Cancer Hospital of Zhengzhou University and Henan Cancer Hospital, Zhengzhou, 450008 China; 2 Department of Hematology, Xinxiang Central Hospital, Xinxiang, 453000 China

**Keywords:** Neutrophil-to-lymphocyte ratio, Peripheral T-cell lymphoma, Prognostic model, Meta-analysis

## Abstract

The neutrophil-to-lymphocyte ratio (NLR) as an inflammatory marker may represent changes between inflammation and host immunity that affect the prognosis of peripheral T-cell lymphoma (PTCL). To comprehensively evaluate the NLR in PTCL, we performed a meta-analysis to investigate the relationship between the NLR and overall survival (OS) and progression-free survival (PFS). PubMed, Embase, Cochrane library, and China National Knowledge Infrastructure (CNKI) were searched for all relevant studies. Hazard ratios (HRs) and 95% confidence intervals (CIs) were obtained from each study. Heterogeneity among the included studies was checked to determine whether fixed or random effects model was used. In total, 8 studies with 921 patients were included for the meta-analysis. High NLR significantly correlated with worse OS (HR = 2.20, 95% CI 1.71–2.83, P < 0.05) regardless of region (Asian or non-Asian), sample size (< 60 or ≥ 60), median age (< 60 or ≥ 60), disease type, or cut-off value (NLR < 3.9 or NLR ≥ 3.9). In terms of PFS, the NLR had no prognostic impact for patients with PTCL (HR = 1.12, 95% CI 0.57–2.20, P = 0.742). Our findings suggest that PTCL patients with high NLR are more likely to have worse OS compared to those with low NLR. Therefore, the NLR can serve as a prognostic marker in PTCL.

## Introduction

Peripheral T cell lymphoma (PTCL) is a group of malignant proliferative diseases originating from mature T lymphocytes, accounting for 10% to 15% of non-Hodgkin's lymphoma (NHL). According to the 2017 WHO classification of Hematopoietic and lymphatic System tumors, PTCL is divided into 23 well-defined subtypes and 6 tentative subtypes [1]. Only a few subtypes are inert, and most of the other subtypes are invasive. The most common subtypes include peripheral T-cell lymphoma, non-otherwise specified (PTCL-NOS), extra-nodal natural killer (NK)/T cell lymphoma, nasal type (ENKTL), angioimmunoblastic T cell lymphoma (AITL), anaplastic lymphoma kinase positive anaplastic large cell lymphoma (ALK+ ALCL) and anaplastic lymphoma kinase negative anaplastic large cell lymphoma (ALK− ALCL). PTCL is a disease with high heterogeneity in biological behavior and clinical manifestation. Currently, there is no consensus first-line chemotherapy regimen. The complete response (CR) rates of traditional anthracycline-based chemotherapy regimens in first-line PTCL therapy ranged from 35.9% to 65.8%, and the 5-year overall survival (OS) rate was 38.5% (95% CI 35.5–41.6%) for all PTCL patients [2]. According to the international T-cell lymphoma project, the 5-year OS of PTCL-NOS was 32% [3]. Long-term OS is less than 35% for most subtypes of PTCL [4].

Some prognostic tools based on clinical variables have been proposed for early prediction of PTCL, including the International Prognostic Index (IPI), the Prognostic Index for PTCLU (PIT), and Prognostic Index of NK/T Cell Lymphoma (PINK). The IPI, which was originally devised for aggressive NHLs in general, is comprised of age, Eastern Cooperative Oncology Group-performance status (ECOG-PS), lactate dehydrogenase (LDH), stage, and extranodal involvement [5]. The PIT was specifically designed for patients with the PTCL-NOS subtype, and includes age, performance status (PS), LDH, and bone marrow (BM) involvement [6]. The PINK includes four independent risk factors: age greater than 60 years, stage III/IV disease, distant lymph-node involvement, and non-nasal type disease [7]. These indices have demonstrated their relevance for treatment response and survival of patients with PTCL. However, some patients with poor prognosis still have not been well identified. Therefore, more effective and feasible biomarkers are urgently needed to predict the prognosis of patients with PTCL.

Studies have shown that peripheral blood inflammatory factors and their associated inflammatory composite index are of great significance for early identification of high-risk patients. In addition, these factors can improve efficacy and prognosis and are expected to be reliable indicators to evaluate the prognosis of patients [8, 9]. The neutrophil-to-lymphocyte ratio (NLR) is an economical, simple, and easily accessible clinical parameter, which compares the degree of the inflammatory response with the immune response, reflecting the relative strength of the host's inflammatory response and immune response. As an inflammatory marker, the NLR has prognostic significance in solid tumors [10–12] and hematologic tumors, such as Hodgkin lymphoma (HL) [13], and multiple myeloma [14], particularly in diffuse large B-cell lymphoma (DLBCL) [15]. However, the prognostic role of the NLR in PTCL has not been widely accepted. To comprehensively evaluate the prognostic significance of the NLR in PTCL, we performed a meta-analysis to investigate the relationship between the NLR and OS and progression-free survival (PFS).

## Materials and methods

### Literature search

A systematic literature search was performed by using database including PubMed, Embase, Cochrane library, and China National Knowledge Infrastructure (CNKI) for all relevant studies. There was no language restriction. The last literature search was updated in June 2021. The following terms were used in the search: “T cell lymphoma”, “neutrophil lymphocyte ratio”. The references in the relevant studies were also screened for possible inclusion.

### Selection criteria

The inclusion criteria were as follows: (a) NLRs were obtained from a hematological test before treatment; (b) the diagnosis of PTCL was confirmed; (c) relationships between the NLR and survival including OS or PFS were investigated, or sufficient data were provided; (d) studies were published as full-text articles in English or Chinese. Studies falling under the following categories were excluded: (a) reviews, meeting abstracts, posters, oral, presentation and duplicate studies, (b) irrelevant studies, (c) animal studies, and (d) studies without sufficient data.

### Data extraction and qualitative assessment

Data were extracted by two independent investigators from eligible studies; discrepancies were resolved by joint discussion. The following information was extracted: first author, year of publication, study location, number of patients, cut-off value, survival outcome, clinicopathological characteristics, and hazard ratios (HRs) with 95% confidence intervals (CIs) for OS and/or PFS. HRs were derived from the results of multivariate Cox regression analysis. The quality of each study was evaluated using the Newcastle–Ottawa Scale (NOS) for cohort studies [16]. Studies with scores of 7 and higher were considered high-quality studies.

### Statistical analysis

The combined HRs and its 95% CIs were used to evaluate the strength of association between the NLR and prognosis. Heterogeneity among included studies was examined by the χ^2^-based Q test and I^2^ test. If there was no significant heterogeneity between studies (P > 0.10 or I^2^ < 50%), the fixed effects model was used. Otherwise, the random effects model was chosen. Odds ratios (ORs) with 95% CIs were used to assess the strength of association between the NLR and clinicopathological parameters. Meta-regression was also performed. Sensitivity analysis was conducted by sequential omission of each of the included studies. Publication bias was tested using Begg's and Egger's, and visual inspection of funnel plot was conducted. All statistical tests were two sided and the significance level was set at 5%. All analyses and graphs were produced using Stata version 15.0 software (Stata, College Station, TX, USA).

## Results

### Literature selection and study characteristics

**Fig. 1:**
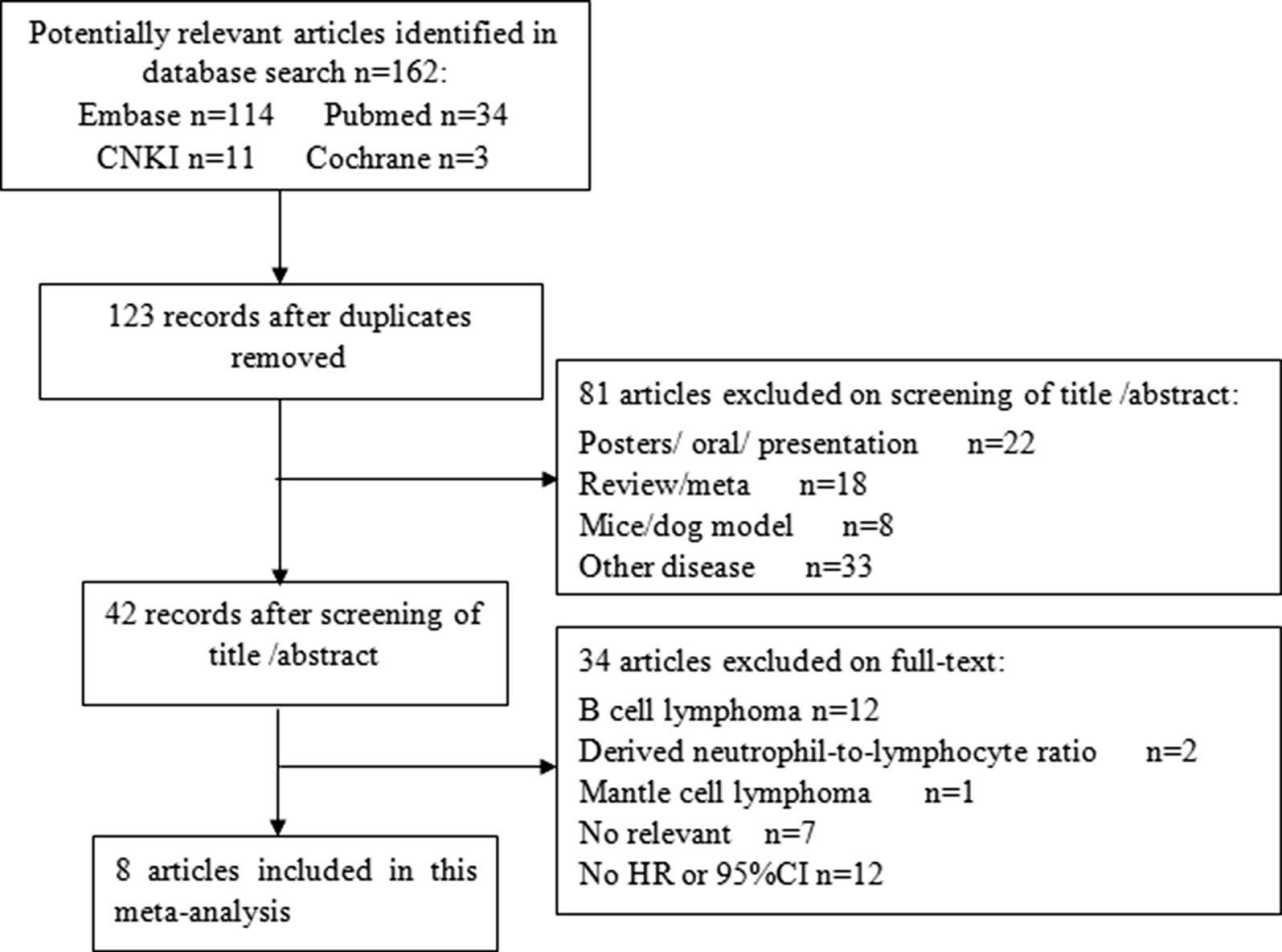
Flowchart of article selection

The flowchart of the literature selection process is shown in Fig. 1. A total of 162 studies were identified through database searching, and 39 records were screened after duplicates were excluded. Then, 81 records were excluded after title and/or abstract review. Subsequently, 42 full-text articles were evaluated for eligibility: 12 articles focused on B cell lymphoma, 2 articles explored the derived NLR, 1 article was about mantle cell lymphoma, 7 articles were not relevant, and 12 articles had no HR or 95% CI. In total, 8 studies with 921 patients were included for the meta-analysis [17–24]. The main characteristics of the included studies are shown in Table 1. The studies were published between 2016 and 2020. Sample sizes ranged from 39 to 191. Five studies were published in English, and 3 studies were published in Chinese. Six studies with 790 patients explored Asian cases, and 2 studies with 131 patients were from Perú. Four studies obtained a cut-off value by receiver-operating characteristic curve (ROC), and others were obtained by median, log-rank test, or reference to other studies. Cut-off values ranged from 3 to 5.5. The median cut-off for the NLR was 3.9. There were 329 patients with PTCL from 4 studies, 39 patients with AITL from 1 study, 553 patients with ENKTL from 3 studies. The median age in the 5 studies with 757 patients was younger than 60 years old, and 2 studies with 87 patients were 60 years old and older.

**Table 1:** Characteristics of studies on PTCL and NLR

Author	Year	Location	Age	Disease type	Sample size	Cut-off value	Method for cut-off value	High-NLR	Low-NLR	OS		PFS		NOS
										HR	95%CI	HR	95%CI	
Yang	2020	China	64 (37–80)	AITL	39	5.43	Median	20	19	6.29	1.76, 22.44	–	–	8
Huang	2020	China	44 (15–82)	ENKTL	184	3.1	ROC	72	112	3.02	1.32, 6.92	–	–	8
Feng	2020	China	55 (15–83)	PTCL	121	3.822	ROC	64	57	1.56	0.86, 2.85	0.62	0.4, 0.94	8
Zhang	2019	China	44 (15–86)	ENKTL	191	5.5	ROC	25	166	1.44	0.73, 2.82	1.42	0.73, 2.76	8
Tan	2019	Singapore/Korea	54 (17.1–86.1)	ENKTL	178	3.5	ROC	59	119	2.08	1.36, 3.18	1.66	1.11, 2.46	8
Choi	2019	Korea	≥ 18	PTCL	77	3	Log-rank test	37	40	1.55	0.64, 3.76	–	–	8
Beltran	2019	Perú	60 (18–83)	PTCL	48	4	NA	13	35	6.2	1.9, 20.9	–	–	8
Beltran	2016	Perú	58 (18–87)	PTCLU	83	4	Refer to the review	29	54	4.73	1.78, 12.6	–	–	8

*ENKTL* extra-nodal natural killer (NK)/T cell lymphoma, nasal type, *PTCL* peripheral T-cell lymphoma, *PTCLU* peripheral T-cell lymphoma unspecific, *AITL* angioimmunoblastic T-cell lymphoma, *ROC* receiver-operating characteristic curve, *NOS* Newcastle–Ottawa Quality Assessment Scale, *OS* overall survival, *PFS* progression-free survival

### NLR and OS, PFS

**Fig. 2:**
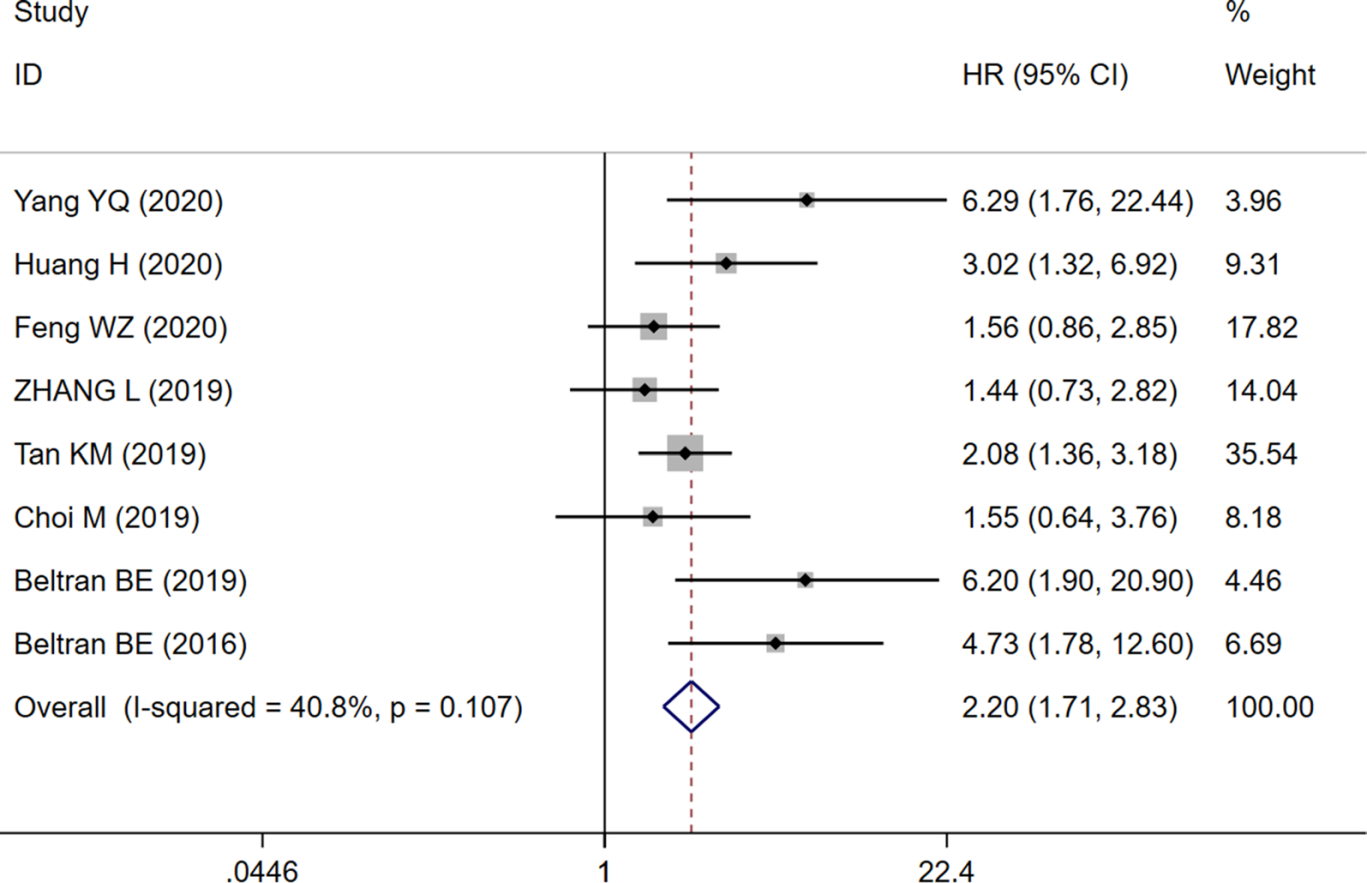
Forest plot of NLR associated with OS

**Fig. 3:**
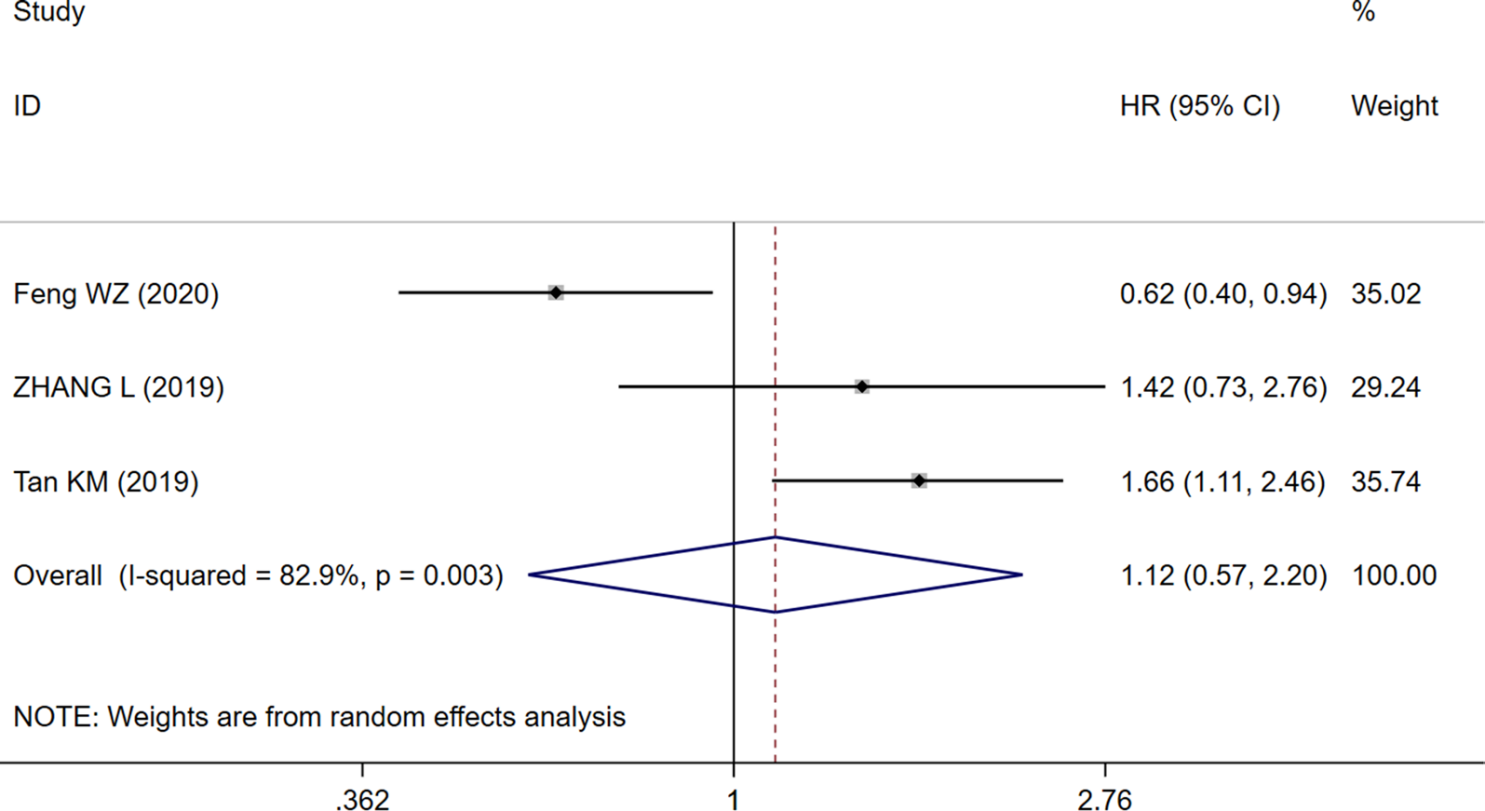
Forest plot of NLR associated with PFS

Eight studies with 921 patients demonstrated a relationship between the NLR and OS in PTCL. Based on the heterogeneity test, there was moderate heterogeneity between the included studies (I^2^ = 40.8%, P = 0.107). Therefore, the fixed effects model was chosen to evaluate the pooled HR. As shown in the Forest plot (Fig. 2), the pooled data demonstrated that high NLR significantly correlated with worse OS (HR = 2.20, 95% CI 1.71–2.83, P < 0.05). HRs for PFS were from 3 studies with 490 patients, and the combined result demonstrated that the NLR had no prognostic impact on PFS for patients with PTCL (HR = 1.12, 95% CI 0.57–2.20, P = 0.742, Fig. 3), and there was significant heterogeneity.

### Subgroup analysis and meta-regression

Subgroup analysis was adopted to explore the causes of heterogeneity for OS analysis. The results shown in Table 2 demonstrated that the NLR remained a significant prognostic marker regardless of region (Asian or non-Asian), sample size (< 60 or ≥ 60), median age (< 60 or ≥ 60), disease type, or cut-off value (NLR < 3.9 or NLR ≥ 3.9). To investigate the effect of various study characteristics on the HR estimates, a meta-regression analysis was conducted with subgroups. No statistical significance was identified regarding the differences in region (P = 0.054), cut-off value (P = 0.231), sample size (P = 0.05), median age (P = 0.366), or disease type (P = 0.232, 0.313).

**Table 2:** Subgroup analysis on PTCL and NLR

Outcome	Variables	No. of studies	No. of patients	I^2^	P-value for heterogeneity	P-value for effects model	HR (95% CI)	P-value for meta-regression
OS	ALL	8	921	40.8%	0.107	< 0.002	2.20 (1.71, 2.83)	
	Region							
	Asian	6	790	16%	0.311	< 0.002	1.97 (1.51, 2.58)	0.054
	Non-Asian	2	131	0	0.732	< 0.002	5.27 (2.47, 11.25)	
	Cut-off value							
	< 3.9	4	560	0	0.586	< 0.002	1.97 (1.46, 2.66)	0.231
	≥ 3.9	4	361	62.7%	0.045	0.002	3.64 (1.60, 8.26)	
	Sample size							
	< 60	2	87	0	0.988	< 0.002	6.24 (2.61, 14.93)	0.05
	≥ 60	6	834	14.20%	0.323	< 0.002	2.00 (1.53, 2.60)	
	Median age							
	< 60	5	757	27.0%	0.241	< 0.002	2.05 (1.55, 2.70)	0.366
	≥ 60	2	87	0.0%	0.988	< 0.002	6.24 (2.61, 14.93)	
	Disease type							
	AITL	1	39	–	–	0.005	6.29 (1.76, 22.43)	
	ENKTL	3	553	0	0.39	< 0.002	2.02 (1.45, 2.81)	0.232
	PTCL	4	329	57.5%	0.07	0.006	2.60 (1.32, 5.13)	0.313
PFS	ALL	3	490	82.9%	0.003	0.742	1.12 (0.57, 2.20)	

*ENKTL* extra-nodal natural killer (NK)/T cell lymphoma, nasal type, *HR* hazard ratio, *PTCL* peripheral T-cell lymphoma, *OS* overall survival, *PFS* progression-free survival, *AITL* angioimmunoblastic T-cell lymphoma

### Association between the NLR and clinicopathological characteristics

Relevant articles among the eight studies in our meta-analysis were enrolled to analyze associations between the NLR and five clinicopathological characteristics (Table 3). The relationship between ECOG-PS and the NLR was examined in six studies with 651 patients, and 7 studies with 726 patients were analyzed for the relationship between LDH level and the NLR. The results demonstrated that high NLR was significantly correlated with elevated LDH (pooled OR: 1.77, 95% CI 1.26–2.48, I^2^ = 32.3%, P = 0.001) and poor ECOG-PS (pooled OR: 0.47, 95% CI 0.24–0.94, I^2^ = 61.9%, P = 0.032). However, the meta-analysis results presented us with no significant association between the NLR and Ann Arbor stage, B symptoms, and IPI score.

**Table 3:** Association between NLR and clinicopathological characteristics

Category	No. of studies	No. of patients	Pooled OR (95% CI)	P-value for effects model	Heterogeneity	
					I^2^ (%)	P-value
Ann Arbor stage (I–II vs. III–IV)	6	686	0.62 (0.26, 1.51)	0.292	62.0	0.022
IPI score (0–2 vs. 3–5)	4	312	0.4 (0.14, 1.11)	0.079	74.0	0.009
B symptoms (+ vs. −)	6	688	1.33 (0.95, 1.88)	0.101	0.0	0.654
LDH (elevated vs. normal)	7	726	1.77 (1.26, 2.48)	0.001	32.3	0.181
ECOG-PS (0–1 vs. 2–4)	6	651	0.47 (0.24, 0.94)	0.032	61.9	0.022

*IPI* international prognostic index, *LDH* lacticdehydrogenase, *ECOG-PS* Eastern Cooperative Oncology Group-performance status

### Sensitivity analysis

**Fig. 4:**
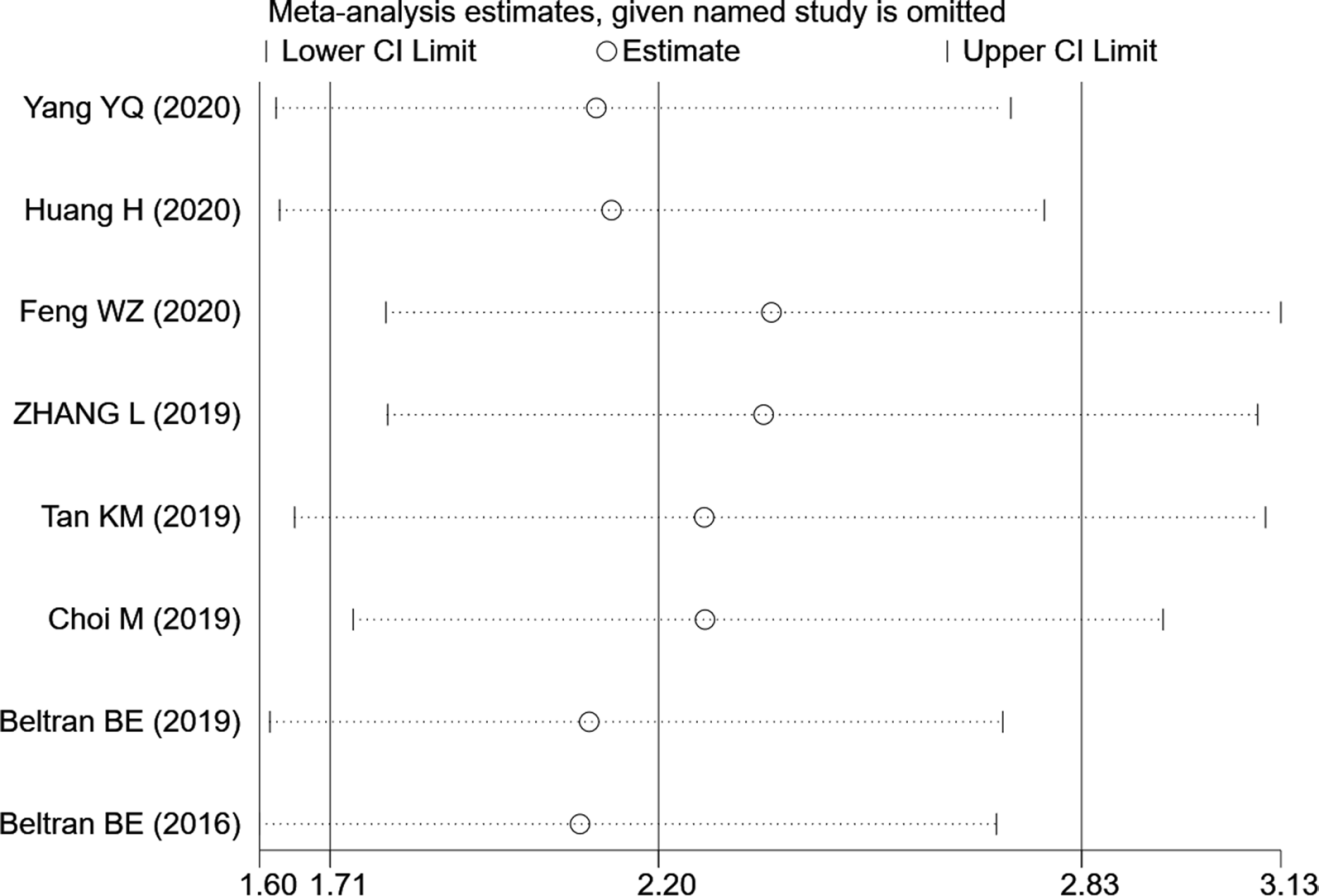
Sensitivity analysis for OS

Sensitivity analysis was conducted by omitting one study at a time and analyzing the remaining studies. The results were not substantially changed, showing the reliability and stability of our results (Fig. 4).

### Publication bias

**Fig. 5:**
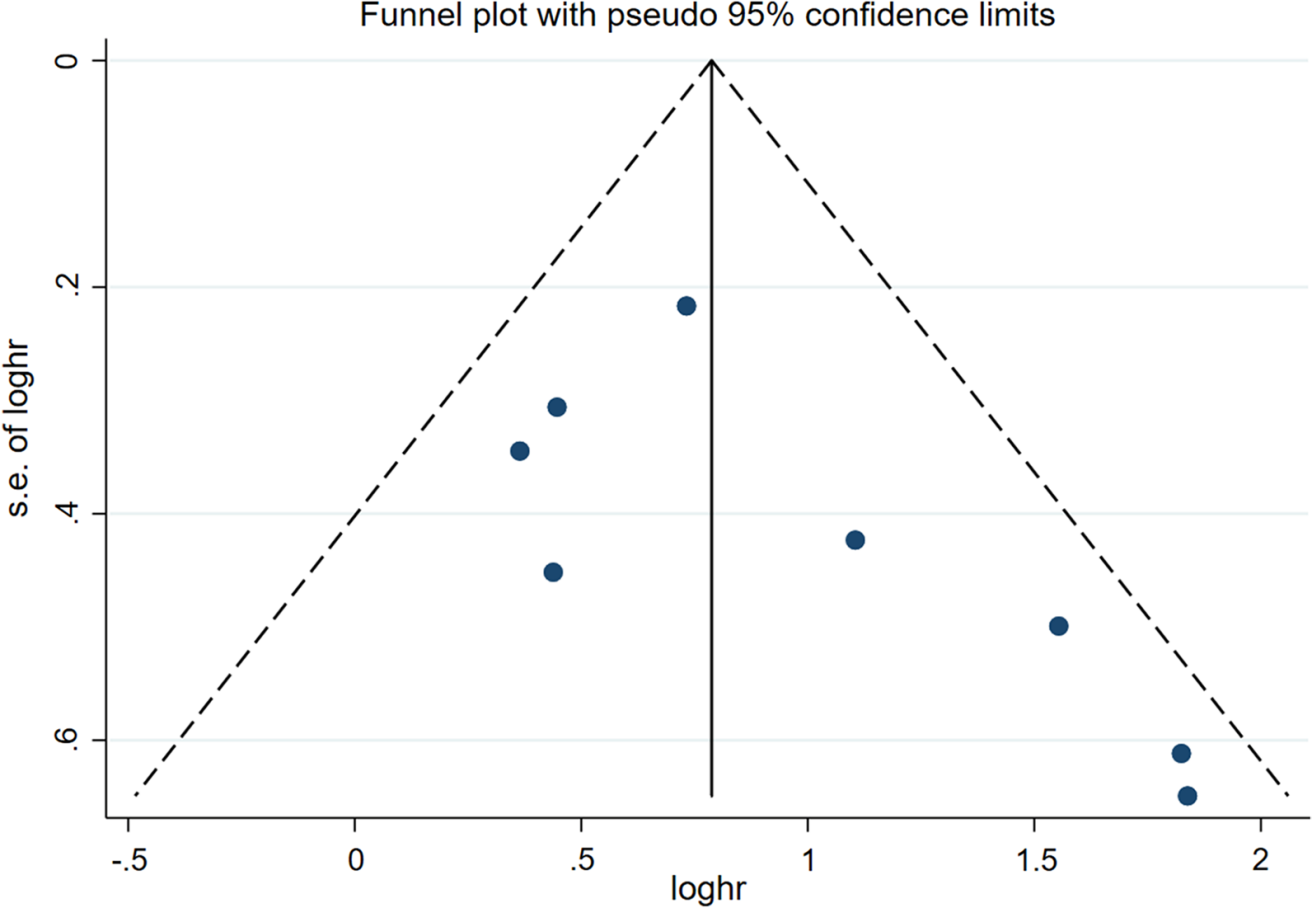
Funnel plot of publication bias for OS

Publication bias was visually assessed using funnel plot. As shown in Fig. 5, the results suggested no significant publication bias for OS on PTCL and the NLR (Begg’s: P = 0.063, Egger's: P = 0.076).

## Discussion

Due to the poor prognosis of PTCL, it is necessary to explore new indicators to further improve prognoses. The systemic inflammatory responses and host immunity triggered by cancer have been regarded as critical components of tumor progression as modification of the tumor microenvironment is largely orchestrated by inflammatory cells [25]. Lymphocytes are an indicator of immune status, systemic inflammatory response can induce lymphocytopenia and then suppress innate immunity. Research shown that lymphocytopenia is associated with the prognosis of ENKTL [26]. Neutrophils contribute to tumor development and progression by providing a favorable environment for tumor growth [27]. Based on studies of the tumor microenvironment, the prognostic role of the NLR in PTCL has attracted more attention in recent years. On multivariate analysis in 59 patients with PTCLs for time to treatment failure (TTF), high NLR was extracted as adverse prognostic factors (HR = 2.25, 95% CI 1.23–4.10, P = 0.008) [28]. Zhao et al. [29] conducted a retrospective study of 213 adult PTCL patients and assessed the prognostic role of the NLR in the CR and survival of PTCL patients, and univariable analysis indicated that the NLR was correlated with worse OS. The results of our meta-analysis confirmed that the NLR can serve as a prognostic marker in PTCL, and high NLR is more likely to indicate worse OS compared to low NLR (HR = 2.20, 95% CI 1.71–2.83, I^2^ = 40.8%), which is the same as that reported in previous studies [22, 23]. However, there was no predictive effect for PFS in our study (HR = 1.12, 95% CI 0.57–2.20, I^2^ = 82.9%), possibly due to the small sample size and high heterogeneity.

Over the past few decades, multiple prognostic models based on clinical indicators have been proposed. ECOG-PS and the LDH level play a certain role in the treatment and prognosis of PTCL, and both are included in the IPI and PIT prognostic models [30]. We analyzed the association between the NLR and five clinicopathological characteristics and found that the NLR was significantly correlated with an elevated LDH and poor ECOG-PS, which further confirmed the prognostic role of the NLR.

Peripheral T and NK/T cell lymphomas, such as nasal ENKTL and adult T cell leukemia/lymphoma (ATLL), are more common in East Asian countries, and the increased incidence in this region is related to ethnic characteristics and the geographical distribution of two virus-associated lymphomas [31–33]. They account for 15% of malignant lymphomas in China and 20% in the Kyushu region of Japan, which is significantly higher than the percentages of Caucasian populations (0–1%) [34]. A retrospective review of 178 patients with biopsy-confirmed ENKTL from the National Cancer Centre of Singapore and the Samsung Medical Center in South Korea show that NLR > 3.5 was a significant predictor for both poor OS (HR = 2.18, 95% CI 1.19–3.99, P = 0.0072) and PFS (HR = 1.74, 95% CI 0.98–3.11, P = 0.0460) in the Singaporean cohort. For the Korean cohort, high NLR was associated with poor OS (HR = 1.94, 95% CI 0.94–3.99, p = 0.0312) but not PFS (HR = 1.30, 95% CI 0.69–2.45, P = 0.372) [21]. Beltran BE et al. [23] analyzed 48 non-Asian patients with a diagnosis of early-stage PTCL between 2001 and 2016, and their stratified analysis demonstrated that the NLR could add prognostic value to the IPI and the PIT scores. Importantly, patients with early-stage aggressive PTCL and NLR ≥ 4 have a dismal prognosis of less than 25% at 3 years. Articles included in our study are mainly from China and South Korea; nonetheless, for both Asian and non-Asian populations, high NLR significantly correlated with worse OS compared to low NLR (HR = 1.97, 95% CI 1.51–2.58 for Asian, HR = 5.27, 95% CI 2.47–11.25 for non-Asian). We also found the same results in ENKTL group (HR = 2.02, 95% CI 1.45–2.81).

In addition to obvious regional differences, the incidence of PTCL was mainly reported in young people (<60 years old) [35–39], and the median age for PTCL in the USA is 62 years, but this varies by subtype [40]. The median age of patients included in our meta-analysis ranged from 44 to 64 years old. Notably, whether the patients were elderly or not, high NLR can be a predictive index for worse OS (HR = 6.24, 95% CI 2.61–14.93 for elderly patients, HR = 2.05, 95% CI 1.55–2.70 for young patients).

Our study is the first meta-analysis to evaluate the prognostic role of NLR in PTCL; however, it does have certain limitations: first, PTCL is a rare malignancy, and the low prevalence of this disease has made it challenging to identify risk factors. Additionally, only 8 studies including 921 patients published in full-text were included in our study; thus, more samples are needed to understand the predictive role of NLR in prognosis, particularly data summarized from non-Asian region. Second, the cut-off value defining a high NLR varied among individual studies, which may have contributed to heterogeneity. Third, the HRs were from multivariate analyses that were adjusted by confounding factors; however, different included studies had different confounding factors. Hence, the merged HRs may have heterogeneity to some extent.

In conclusion, our findings suggest that PTCL patients with high NLR are more likely to have worse OS compared to those with low NLR. Therefore, NLR can serve as a prognostic marker in PTCL.

## Data Availability

The datasets used in this study are available from the corresponding author upon reasonable request.
